# Periodical Lameness in the Horse

**Published:** 1896-06

**Authors:** 


Periodical Lameness in the Horse. T. A. L. Beel reported a case of a Geldersland mare, about eight years old, which showed periodical lameness every ten days. The first time about ten days before, while being driven. Although she had come out from the stall sound and well, the left hindleg began gradually to be moved less nimbly toward the front, and after a while she dragged it over the ground, while a sweat broke out over the whole body. After resting awhile the mare regained control of her limb. On the way back the same thing occurred near the stable. After this the mare was given a rest of some days, and no lameness was noticed until she was driven again. The interval between the harnessing and the appearance of the lameness was at first about a half-hour, afterward fifteen minutes. For the purpose of witnessing the onset of the lameness, being too far away from the house to make a daily examination, I had the patient hitched up in the threshing-machine. After a quarter of an hour a trailing of the near hindleg showed itself plainly, and developed quickly into a dragging, after which the animal began to sweat over the whole body. The mucous membranes became deep red, the eye had a staring look, and the mare could not move from the spot. The near hindleg did not perspire at all; on the inner surface especially it was as cold as ice, and was held stretched out toward the rear and turned outward. Pricking it with a pin was felt to a less degree than should have been the case. On closer examination an increased pulsation of the basilar artery could be noticed far toward the front. I could not convince myself, however, of any change in the bloodvessel behind the place of increased pulsation. Presumably pulsation was very much retarded, inasmuch as the horse was very fat. During the attack, which lasted five minutes, the patient continued to look around at its leg, probably on account of the "sleepy feeling." Gradually the horse began to move again, but the attacks recurred then more quickly. Apparently it was a case of stagnation of arterial blood from the muscular system, especially from the near hindleg, the result of obliteration (reduction to a low state) of the artery leading to it. My prognosis was unfavorable, and treatment (in order to please the owner) consisted in perfect rest, smearing the rump and hindlegs with a light liniment, together with the application of Priesnitz's poultices. By the increased flow of blood toward the rear parts the production of a collateral circulation of blood was furthered. The patient was allowed to rest until the beginning of April, and then seven months later put to work, without a return apparently of the trouble, recovery having taken place.—*Tijdschrift voor Veeartseuijkunde en Veeteelt.*, January, 1896.

